# Developmental Effects of Perfluorononanoic Acid in the Mouse Are Dependent on Peroxisome Proliferator-Activated Receptor-Alpha

**DOI:** 10.1155/2010/282896

**Published:** 2010-09-27

**Authors:** Cynthia J. Wolf, Robert D. Zehr, Judy E. Schmid, Christopher Lau, Barbara D. Abbott

**Affiliations:** ^1^Toxicology Assessment Division, National Health and Environmental Effects Research Laboratory, Office of Research and Development, US Environmental Protection Agency, Research Triangle Park, Durham, NC 27711, USA; ^2^Research Core Unit, National Health and Environmental Effects Research Laboratory, Office of Research and Development, US Environmental Protection Agency, Research Triangle Park, Durham, NC 27711, USA

## Abstract

Perfluorononanoic acid (PFNA) is one of the perfluoroalkyl acids found in the environment and in tissues of humans and wildlife. Prenatal exposure to PFNA negatively impacts survival and development of mice and activates the mouse and human peroxisome proliferator-activated receptor-alpha (PPAR*α*). In the current study, we used PPAR*α* knockout (KO) and 129S1/SvlmJ wild-type (WT) mice to investigate the role of PPAR*α* in mediating PFNA-induced *in vivo* effects. Pregnant KO and WT mice were dosed orally with water (vehicle control: 10 ml/kg), 0.83, 1.1, 1.5, or 2 mg/kg PFNA on gestational days (GDs) 1–18 (day of sperm plug = GD 0). Maternal weight gain, implantation, litter size, and pup weight at birth were unaffected in either strain. PFNA exposure reduced the number of live pups at birth and survival of offspring to weaning in the 1.1 and 2 mg/kg groups in WT. Eye opening was delayed (mean delay 2.1 days) and pup weight at weaning was reduced in WT pups at 2 mg/kg. These developmental endpoints were not affected in the KO. Relative liver weight was increased in a dose-dependent manner in dams and pups of the WT strain at all dose levels but only slightly increased in the highest dose group in the KO strain. In summary, PFNA altered liver weight of dams and pups, pup survival, body weight, and development in the WT, while only inducing a slight increase in relative liver weight of dams and pups at 2 mg/kg in KO mice. These results suggest that PPAR*α* is an essential mediator of PFNA-induced developmental toxicity in the mouse.

## 1. Introduction

Perfluorinated alkyl acids (PFAAs) are a family of chemicals that have a fatty acid-like carbon backbone saturated with fluorine and a functional group at the end. They are surfactants used in many consumer and industrial applications such as waterproofing and stain repellent on clothing, carpets, and other fabrics, oil repellent on food packaging, fire-fighting foams, paints, adhesives, hydraulic fluids, among others [1–4]. Their widespread use in consumer and industrial products is matched by their global presence in the environment [2, 5, 6] and in wildlife and humans [7–12]. The ubiquitous presence of these chemicals, especially in human sera, has led to concern about their safety. The two most common PFAAs, perfluorooctanoic acid (PFOA) and perfluorooctane sulfonate (PFOS), have been found in laboratory animals to induce hepatotoxicity, carcinogenicity, immunotoxicity, disruption of thyroid hormone levels, and developmental effects including prenatal and neonatal mortality, stunted mammary gland development, developmental delay, and reduced body weight (reviewed [6, 13]). Although the manufacture of PFOS was phased out in the United States and the manufacture of PFOA is being phased out, alternative PFAAs have been marketed for use.

Perfluorononanoic acid (PFNA) is a 9-carbon member of the PFAA family found in the environment and in serum at levels much lower than those of PFOA or PFOS. Nevertheless, levels of PFNA in human serum have risen in the last several years and currently stand at around 1 ng/ml [7, 14]. Its presence in human serum has been shown to correlate with PFNA ingested from food and water [15, 16]. Few studies have investigated its toxicity. In vitro studies found PFNA to be cytotoxic in HCT-116 cells [17], and hepatotoxic [18]. PFNA was also found to be immunotoxic *in vivo* [19, 20]. More recently, PFNA was found to induce developmental toxicity in mice when administered throughout the gestational period [21]. Adverse effects of exposure to PFNA during gestation include reduced postnatal survival at 5 mg/kg/day, delayed eye opening, delayed puberty, increased liver weight, and reduced body weight at 3 and 5 mg/kg/day. 

One of the mechanisms implicated in the toxicity of the PFAAs is the activation of peroxisome proliferator-activated receptor-alpha (PPAR*α*). PPAR*α* is a nuclear receptor that plays a role in regulating lipid and glucose homeostasis, cell proliferation and differentiation, and inflammation [22]. PPAR*α* activation is thought to be responsible for PFOA-induced hepatotoxicity in rodents [23] and certain immunotoxic effects [20, 24, 25]. In addition to PFOA, a number of other PFAAs activate PPAR*α* *in vitro* [26–28]. PPAR*α* may mediate developmental processes, since PPAR*α* is present during murine development [29]. The developmental toxicity of PFOA in mice, including neonatal lethality, delayed eye opening, and reduced body weight, was found to be dependent on PPAR*α* [30] although developmental toxicity of PFOS was not [31]. PPAR*α* may also mediate PFNA effects. Evidence of PPAR*α* activation was found in livers of mice exposed to PFNA during fetal development [21]. PFNA also activates PPAR*α* *in vitro* and was the most effective of the PFAAs tested in activating both human and murine PPAR*α* in transfected COS-1 cells [28]. It is therefore logical to postulate that the developmental toxicity of PFNA, like PFOA, may also be dependent on PPAR*α*.

In the current study, we sought to determine whether PFNA-induced developmental toxicity in the mouse requires expression of PPAR*α*. Pregnant 129S1/SvlmJ wild-type (WT) and PPAR*α* knockout (KO) mice were given PFNA during gestation, and indices of fertility and neonatal development, along with serum levels of PFNA, were evaluated. We report that the developmental effects of PFNA including pup survival, eye opening, and body weight are dependent on PPAR*α* and that hepatomegaly is primarily PPAR*α* dependent but may utilize other pathways as well.

## 2. Materials and Methods

### 2.1. Animals

Male and female wild-type (WT) 129S1/SvlmJ mice (stock no. 002448) and PPAR*α* knockout (KO) mice on a 129S1/SvlmJ background (Ppara-tm1Gonz/J, stock no. 003580) were obtained from Jackson Laboratories (Bar Harbor, ME). WT and KO mice were kept in breeding colonies in the EPA Reproductive Toxicology Facility, Durham, NC. Colony animals were group housed by sex in Tecniplast cages (Tecniplast USA, Exton, PA) with Beta-chip hardwood bedding (Northeastern Products, Warrensburg, NY) in a closed ventilation system, provided pelleted mouse chow (LabDiet 5001, PMI Nutrition International LCC, Brentwood, MO) and tap water *ad libitum*, and kept in an atmosphere of 68–74°F and 40–60% humidity with a 12-hour light-dark cycle. All animal studies were conducted in accordance with guidelines established by the USe EPA ORD/NHEERL Institutional Animal Care and Use Committee. Procedures and facilities were consistent with the recommendations of the 1996 NRC “Guide for the Care and Use of Laboratory Animals”, the Animal Welfare Act, and Public Health Service Policy on the Humane Care and Use of Laboratory Animals.

### 2.2. Study Design and Protocol

The study was conducted in four blocks with WT and KO represented in each block. WT and KO females were mated overnight to males of their respective strain, one mating pair per cage. Females were checked for vaginal plugs the following morning and plug positive animals were weighed, randomly assigned to treatment groups, and housed individually in regular ventilated polypropylene cages. Day of plug was considered gestational day (GDs) 0. Animals of each strain were weighed and dosed by oral gavage once daily on GD 1–18 with either water (vehicle control: 10 ml/kg) or PFNA (CAS# 375-95-1; 97% pure; Aldrich, St. Louis, MO) at 0.83, 1.1, 1.5, or 2.0 mg/kg, based on previous studies with PFNA and PFOA [21, 30]. Dosing solutions were prepared by dilution, fresh daily immediately before dosing. At term, adult females were checked twice daily for the presence of pups. Adult females with pups or those were pregnant were called dams. Day of birth was considered postnatal day (PND) 0. Dams and pups were monitored on a daily basis. The numbers of live and dead pups were recorded twice daily, and live pups were weighed by sex on postnatal days 0, 1, 2, 3, 7, 10, 14, and 21 (weaning). Pups were monitored for eye opening daily from PND 11 until all eyes were open. Eye opening is described as the percentage of pups per litter having both eyes completely open and was identified by technicians trained by demonstration and protocol to eliminate subjectivity. All animals on study were sacrificed for necropsy on PND 21 (42 days postcoitus for nonpregnant adult females). Body and liver weights were measured from each adult female and from 2 pups per litter. Blood was collected from each dam individually and from all pups pooled by litter. Serum was extracted and stored at −20°C. Uteri were collected from all adult females, stained with 2% ammonium sulfide, and uterine implantation sites were counted [32].

### 2.3. Serum Analysis of PFNA

Analysis of PFNA in serum was performed using a modification of a method previously described in [33]. For the current study, 25 *μ*l of serum was placed in a 6 ml polypropylene tube, deproteinized with 1 ml of 0.1 M formic acid, and vortexed. Two hundred *μ*l of this mixture was then transferred to a fresh 6 ml polypropylene tube and spiked with 2 ml acetonitrile containing 25 ng/ml ^13^C_9_-PFNA (Cambridge Isotope Laboratories, Inc., Andover, MA). The tube was vortexed for 20 minutes and then centrifuged for 3 minutes at 3500 rpm to precipitate proteins or other residue. Two hundred *μ*l of the supernatant was then transferred to a 500 *μ*L polypropylene autosampler vial and mixed with 200 *μ*l of 2 C mM ammonium acetate for HPLC/MS-MS analysis. Solutions were analyzed using an Agilent 1100 high-performance liquid chromatograph (Agilent Technology, Palo Alto, CA) coupled with an API 3000 triple quadrupole mass spectrometer (LC/MS-MS; Applied Biosystems, Foster City, CA). Ten *μ*l of solution was injected onto a Luna C18(2) 3 × 50 mm, 5 *μ*m column (Phenomenex, Torrance, CA) using a mobile phase consisting of 30% 2 mM ammonium acetate solution and 70% acetonitrile. Peak integrations and areas were determined using Analyst software (Applied Biosystems Version 1.4.1). For each analytical batch, matrix-matched calibration curves were prepared using mouse serum spiked with varying levels of PFNA (Aldrich, St. Louis, MO). For quality control, check standards were prepared by spiking large volumes of mouse serum at several arbitrary levels. Check standards were stored frozen and aliquots analyzed with each analytical set. In addition, control mouse serum samples were fortified at two or three levels in duplicate with known quantities of PFNA during the preparation of each analytical set. Duplicate fortified and several check standards were run in each analytical batch to assess precision and accuracy. The limit of quantitation (LOQ) was set as the lowest calibration point on the standard curve. Analytical batches were considered to be acceptable if matrix and reagent blanks had no significant PFNA peaks approaching the LOQ, the standard curve had a correlation coefficient >0.98, and all standard curve points, fortified, and check samples were within 70%–130% of the theoretical and previously determined values, respectively.

### 2.4. Data Analysis

Maternal pregnancy, neonatal development, and necropsy data were analyzed in GraphPad Prism (version 4; San Diego, CA). Individual means (maternal data) or litter means (pup data) and standard errors were obtained by dose group and strain and analyzed by ANOVA. Pairwise *t*-tests were computed within ANOVA to compare individual treatment groups to relevant control groups within strain. A Bonferroni multiple-comparison adjustment was used when appropriate. Linear regression analysis was performed on liver data to detect dose-related trends. Pregnancy rate was analyzed using chi-square trend analysis. Litter loss is described as dams that had full litter resorption (FLR, uterine implants but no pups at birth) or whole litter loss (WLL, only dead pups at birth). Litter loss was examined for treatment effect using chi-square analysis. Serum data were analyzed in SAS for Windows v9.1 (SAS, Cary, NC). Analyses were performed separately for adult females and for pups. Adult females were further separated into pregnancy and lactation status (with live pups or with no live pups including nonpregnant and litter loss). A subset of dams matched with their pups was used to determine differences in levels of PFNA between dams and pups. Where variances were heterologous, data were log10 transformed to calculate means and standard errors and analyzed by ANOVA to investigate effects of treatment, strain (WT, KO), and block. When treatment differences were found by ANOVA, pairwise t-tests between treatment groups were calculated within each strain and separately by dams or pups, using Tukey-Kramer adjustment for multiple comparisons where appropriate.

## 3. Results

### 3.1. Maternal Pregnancy Outcome and Gestational Body Weight

Daily maternal body weight and maternal weight gain from GD 1 to GD 18 were not affected by gestational PFNA exposure. Implantation and total litter size (live and dead pups) at birth were not affected in either strain. However, the number of live pups at birth was significantly reduced in the WT strain at 1.1 (*P  *< .05) and 2.0 (*P  *< .001) mg/kg PFNA (Table 1) while being not significant at 1.5 mg/kg. Percent litter loss was not significantly altered in any dose group in KO or WT although there was a modest but insignificant increase in litter loss in the WT (Table 1). In each dose group in the KO, only 1 or 2 dams had FLR or WLL while, in the WT group, exposed to 2 mg/kg PFNA, 4 dams had FLR and 2 had WLL (35% litter loss). Most dams with FLR did not gain weight comparable to the pregnant dams that delivered litters, which suggests that FLR occurred early in gestation. Dams with WLL gained weight and carried to term, but it cannot be determined by our protocol whether these pups died prior to delivery or soon after delivery. Pregnancy rate, the percentage of plugged mice that had uterine implants, was reduced in treated KO groups (*P* < .001) but not in WT groups, suggesting that PFNA may have interfered with implantation when PPAR*α* was not functional.

### 3.2. Pup Survival, Development, and Body Weight

**Figure 1:**
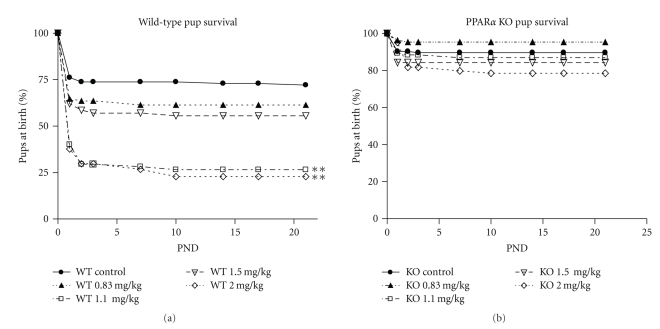
Effects of prenatal exposure to PFNA on survival of wild-type (WT) and PPAR*α* knockout (KO) mouse pups. Data represent litter means ± SEM of the percent of the litter alive on postnatal days 0–3, 7, 10, 14, 17, and 21. Survival was reduced in WT pups by 1.1 and 2.0 mg/kg PFNA on GDs 1–18. Asterisks denote a significant difference (*P* < .001) found by ANOVA and Bonferroni's test for multiple comparisons. PND: postnatal day.

The reduced viability of pups at birth in the WT at 1.1 and 2 mg/kg continued through the postnatal period. Survival of WT pups from birth to weaning (PND 21) was greatly reduced at 1.1 (*P *< .05) and 2 (*P *< .001) mg/kg PFNA (Figure 1). By PND 21, survival of pups in the WT 1.1 and 2.0 mg/kg groups was reduced to 36% and 31%, respectively. In contrast, survival was not affected in the KO at any dose.

**Figure 2:**
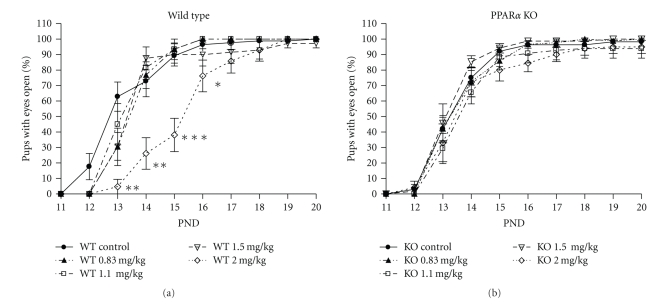
Effect of prenatal exposure to PFNA on the percent of eyes open on postnatal days 13–16 in wild-type (WT) and PPAR*α* knockout (KO) mouse pups. Data represent litter means ± SEM of the percent of the litter with pups having both eyes fully open. A reduction in the percent of eyes open was found in the WT pups exposed to 2 mg/kg PFNA. Differences were found by ANOVA and Bonferroni's test for multiple comparisons. Asterisks denote a significant difference (*P < .05, **P < .01, ***P < .0001). PND: postnatal day.

Eye opening was used as a marker of postnatal development. The mean day of eye opening in the controls was PND 13.7 ± 0.3 in WT and PND 13.9 ± 0.2 in KO. The mean day of eye opening was significantly delayed at 2 mg/kg PFNA in the WT by two days, to PND 15.8 ± 0.2 (*P* < .01), but not at any other dose. In contrast, the mean day of eye opening was not affected at any dose in KO. The percent of eyes open on PNDs 13, 14, 15, and 16 was also significantly reduced in the WT at 2 mg/kg PFNA while being not affected in the KO (Figure 2). 

**Figure 3:**
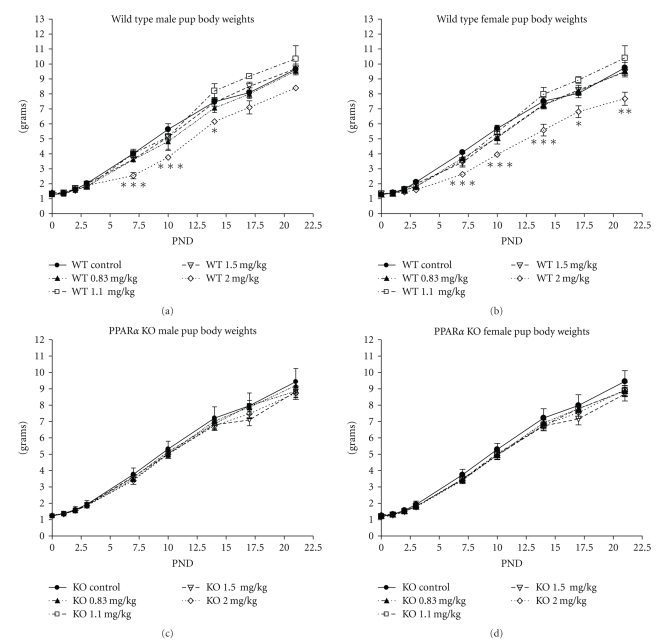
Effects of prenatal exposure to PFNA on postnatal body weights of wild-type (a, b) and PPAR*α* knockout (c, d) male (a, c) and female (b, d) pups. Data represent litter means ± SEM on postnatal days 0–3, 7, 10, 14, 17, and 21. Body weights of WT pups were reduced by 2 mg/kg PFNA on postnatal days 7, 10, and 14, in male pups and days 7–21 in females. No effect on body weight was found in KO pups. Significant differences were found by ANOVA, and differences between groups were found by Bonferroni's test for multiple comparisons. Asterisks denote a significant difference (*P < .05 by *t*-test, **  *P* < .01, ***  *P* < .001).

Pup birth weight was not affected by any dose of PFNA in WT or KO, either in males or females (Table 2). Although pup body weight was not different among groups at birth, pup body weight was reduced in both male and female WT pups in the 2 mg/kg group at several time points during the postnatal period, beginning at PND 7 and including weaning (Figure 3). Weight gain during this period was reduced in WT female pups from 8.52 g in controls to 6.35 g in the 2 mg/kg group (*P* < .001), but not in male. In contrast, body weight and weight gain were not affected at any age at any dose level in the KO (Figure 3).

### 3.3. Liver Weight and Body Weight at PND21

**Figure 4:**
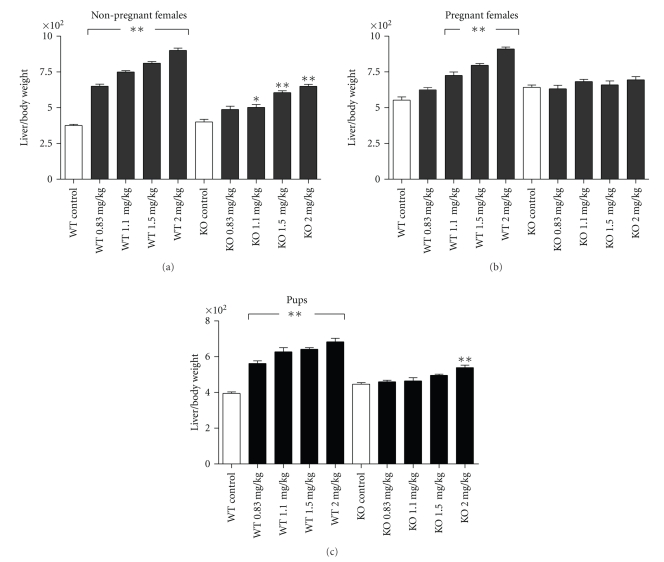
Effects of gestational exposure to PFNA on relative liver weight of the wild-type (WT) and PPAR*α* knockout (KO) nonpregnant adult female (a) dams (b) and pups (c) Measurements were taken on all individual adult females and on two pups per litter at weaning (i.e., 23 days after last dose or postnatal day 21). Data represent means or litter means ±SEM. Relative liver weight was calculated as the absolute liver weight/body weight ×100 for each data point. Relative liver weight was increased by PFNA exposure in both pregnant and non-pregnant adults and pups in all treated groups in the WT while only in the nonpregnant adult KO at 1.1 to 2 mg/kg and in the KO pup at 2.0 mg/kg. Significant differences were found by ANOVA, and differences between groups were found by Bonferroni's test for multiple comparisons. Asterisks denote significant differences compared to controls (*P < .05, **  *P* < .001).

Absolute liver weight was increased in a dose-dependent fashion in all PFNA-treated groups in WT adult females, regardless of prior pregnancy status. In KO adult females, however, liver weight was not affected by PFNA in dams but was increased in the 1.5 and 2.0 mg/kg groups in the nonpregnant adult (Table 3). In addition, among the nonpregnant adults, the dose dependent increase in liver weight was lower in KO compared to WT (*P* < .0001, by regression analysis). Similarly, relative liver weight was increased in a dose-dependent fashion in all treated groups in the WT (*P* < .001), regardless of pregnancy history, and in 1.1 mg/kg and higher doses in the nonpregnant KO (Figure 4). In KO adults that had been pregnant, relative liver weight was unaffected. Body weight at necropsy was generally unaffected by dose or strain (Table 3). Absolute liver weight was increased in all PFNA dose groups in WT pups but was unaffected in KO. Relative liver weight was increased in all dose groups in WT pups but in only the highest dose group, 2 mg/kg, in KO (Figure 4). Body weight was not reduced in KO pups at any dose. Pup body weight was reduced in WT at 2 mg/kg only (Table 3).

### 3.4. Serum PFNA Levels

PFNA was detected in serum of all animals (Table 4). PFNA levels were significantly higher in PFNA-treated mice at every dose level compared to controls (*P* < .0001) and levels increased in a dose-dependent fashion. Serum PFNA levels were higher in adult females with no live pups (regardless of pregnancy) compared to adults with live pups by *P* < .001 (KO) and *P* < .005 (WT). PFNA levels were also higher in pups compared to their dams, based on a subset of dams matched to their existing pups at weaning (KO, *P* < .0001; WT, *P* < .005). In all dams with nursing pups, PFNA levels were lower in KO compared to WT (*P* < .001) while, in pups, PFNA levels were higher in KO compared to WT (*P* < .0001; Table 4).

## 4. Discussion

Perfluorononanoic acid (PFNA) has recently been shown to induce developmental toxicity and liver enlargement in mice [21], as do other perfluoroalkyl acids. The purpose of the current study was to determine whether these effects are dependent on PPAR*α*, using the 129S/SvlmJ PPAR*α* knockout (KO) mouse model. Gestational exposure to PFNA reduced neonatal survival and body weight through the weaning period, delayed eye opening, and increased absolute liver weight in the WT offspring at doses as low as 0.83 mg/kg/day. By contrast, these effects were not seen in KO offspring. These findings demonstrate that PFNA is a developmental toxicant and its effects are dependent on expression of PPAR*α*. 

This pattern of reduced survival, body weight, delayed development, and increased liver weight is common to most perfluoroalkyl acids (PFAAs) studied thus far. These effects have been reported in rodents for PFOA [30, 34], PFOS, [31, 35, 36], and PFNA [[21, 37], Das, 2010 #389], with a few specific differences that may be due to strain, dosing regimen, and the chain length and functional group of the PFAA. Such studies also obtained effects in offspring at dose levels that are not maternally toxic [30, 31, 34, 35, 38], as shown here. Also common to the current and previous studies, the liver was the most sensitive target tissue, with effects on liver weight seen in both WT dam and pup from the lowest dose level of PFNA used in the study, 0.83 mg/kg, and higher. Reduced survival, body weight, and delayed eye opening in pups were also sensitive endpoints, inducing effects at the next higher dose levels, 1.1 and/or 2.0 mg/kg. Survival and number of live pups at birth were compromised at 1.5 mg/kg, but the values did not reach statistical significance. The reason for this finding is unclear. The serum PFNA concentrations and the liver weight in the pups in this dose group were in the expected ranges for a linear dose-response curve, suggesting proper dose preparation and administration for 1.5 mg/kg. In addition, the “*n*” of 12 litters in this treatment group was comparable to that of other dose groups, so it seems unlikely that the outcome is related to a low statistical power. Thus, the lack of consistent effect on survival cannot be explained and may simply reflect biological variability. Nonetheless, all developmental endpoints were clearly PPAR*α* dependent. The dependence of the developmental effects of PFNA on PPAR*α* is not unique, as this has also been demonstrated previously for PFOA [30]. However, not all PFAAs depend upon PPAR*α* to induce developmental effects. The developmental effects of PFOS, for example, were not found to be dependent on PPAR*α* [31]. This may be due to the sulfonated head group of PFOS, and thus PPAR*α* dependence may be a feature of the perfluorocarboxylic acids. 

Mode of action differences between the perfluorinated carboxylic acids, PFOA and PFNA, and the sulfonate PFOS may also be evident in the pattern of neonatal loss observed following exposure to these compounds. PFNA exposure in WT mice resulted in a drastically reduced number of viable pups at birth with a continued loss of pups within the first few days, followed by a gradual loss until PND 10. Similarly, PFOA induced a sudden loss of viable pups within the first few days of life, with a gradual loss over 10 days in CD-1 mice [38] and 14 days in the 129S/SvlmJ strain [30]. In contrast to our study, PFNA in CD-1 mice induced a gradual loss of pups over the course of 12 days with no significant loss at birth [21]. This difference may be due to the increased sensitivity of the 129S/SvlmJ strain. Although survival curves for PFOA and PFNA can follow a course of up to 10–14 days, *in utero* exposure to PFOS results in a sudden loss of viability in pups within the first few hours after birth through postnatal day 2 in the rat [35, 36]. These pups were observed to be in respiratory distress and displayed poor inflation of the lungs [36, 39] although the precise mechanism has not been found. This two day loss of pups after exposure to PFOS was observed in the 129S/SvlmJ strain as well, and only in KO did a few more die as late as PND 10 [31]. Therefore, in neonates, PFNA may be utilizing the same mechanism of action as other perfluorinated carboxylates while sulfonates such as PFOS utilize another. 

In the liver, there appear to be PPAR*α*-independent as well as PPAR*α*-dependent events in response to PFNA. PFNA was found to increase relative and absolute liver weight in the WT adult, but to a lesser extent in the nonpregnant KO, and not at all in the pregnant KO adult. The lack of effect on liver weight in the pregnant KO may suggest that the effects of PFNA on liver weight in adult KO mice are modest and were masked by the increase in liver weight due to pregnancy. The attenuated response in the KO liver compared to the WT liver is more obvious in the pup and may imply a separate, less efficient mechanism independent of PPAR*α*. Similarly, less robust effects on liver weight in KO compared to WT mice were observed after exposure to PFOA [30]. Histopathological examination of those livers revealed a difference in histology of treated KO livers compared to treated WT livers [40], suggesting a different mechanism in KO mice. Other pathways suggested have included constitutive androstane receptor (CAR) and pregnane X receptor (PXR) [41–43], both present in humans. Therefore, PFNA may primarily utilize PPAR*α* to increase liver weight while relying upon other pathways in the absence of PPAR*α*. Involvement of PPAR*α* in the liver may be a mechanism utilized by other PFAAs, since perfluorobutyrate also increased liver weight and induced hepatocyte hypertrophy dependent on PPAR*α* [44]. Relevance of the PPAR*α* mechanism to humans has been criticized primarily based on the lower number of these receptors in the liver of human versus mouse. However, PPAR*α* is implicated here in the developmental effects of PFNA as well, and the etiology of PPAR*α* in other tissues of the embryo, fetus and neonate of the human and the mouse that are involved in gross development has not been fully determined. Therefore, the possibility of relevance of PPAR*α* to a human response to PFNA cannot be dismissed.

The levels of PFNA in the serum of pups, nursing dams, and adult females with no pups illustrate some interesting findings. First, the dose-dependent serum levels of PFNA in all groups of animals reflect the dose-dependent effects observed in dams and pups. Second, the effects observed in WT pups were not due to higher concentrations of PFNA in their system, since serum levels of PFNA were actually lower in WT pups than in KO pups at all doses. Conversely, the general lack of developmental effects in KO pups was not due to impaired pharmacokinetic distribution of PFNA to the pup. Another important observation is the possibility of substantial transfer of PFNA from dam to pup through milk. PFNA can enter milk, as evidenced by the finding of PFNA in the milk of humans [45–47], rats [48], and mice [49]. The lower serum PFNA levels in lactating dams compared to nonlactating adult females at weaning suggest an elimination of PFNA from the dams through placental transfer and through the milk. In addition, PFNA levels were elevated in pups compared to their mothers. This has also been reported for PFOA, in which an increased body burden was observed in the pups from birth to postnatal day 8 [49]. However, the contribution of placental versus lactational transfer of PFNA cannot be determined by the design of this study. Serum levels of PFNA in this mouse model were much higher than those of humans [7] but were measured to compare with the physiological effects observed and not to compare to human levels. 

PFNA was found in this study to be a liver and developmental toxicant comparable in strength to other PFAAs, as adverse responses were elicited at maternal doses as low as 0.83 mg/kg. In the CD-1 mouse, PFNA appears to be more potent than PFOA. PFNA reduced CD-1 pup survival at 5 mg/kg/day, compared to10 mg/kg/day by PFOA, and delayed eye opening at 3 mg/kg/day compared to 5 mg/kg/day by PFOA [21, 34, 37, 38]. *In vitro* analysis of PPAR*α* activation shows PFNA to be more potent than PFOA as well [28]. The 129S strain used in the current study was used as an animal model for investigating mechanisms of action rather than for relative potency, as toxicity and PBPK data are lacking and this strain appears to be more sensitive to PFAAs. It is also clear that PFNA is more potent than PFOS. PFOS induced a 50% reduction in survival in CD-1 offspring at 10 mg/kg/day [35] and at 8.5 mg/kg/day in 129S/SvlmJ mice [31] whereas PFNA reduced survival at 1.1 mg/kg/day in the current study or 5 mg/kg in CD-1 mice. Given the lower activity of the sulfonated PFAA compared to the carboxylated PFAA on PPAR*α* *in vitro* [28], lower potency *in vivo* may be expected for other sulfonated PFAAs as well.

## 5. Conclusion

In summary, PFNA is a developmental toxicant in mice, and the developmental effects are dependent upon expression of PPAR*α*. The general pattern of effects observed in the mouse after gestational PFNA exposure mirrors the effects of other PFAAs, most closely that of PFOA. In addition, the differential response to PFNA in the livers of WT and KO adult females suggests a PPAR*α*-dependent mode of action for increased liver weight, although additional pathways and mechanisms appear to be involved.

## Figures and Tables

**Table 1:** Effects of gestational administration (GD 1–18) of PFNA to wild type and PPAR*α*KO mice on maternal weight and reproductive outcomes.

Strain	Dose (mg/kg/day)	No. of pregnant^a^	Maternal Weight Gain GD1–18^b^ (g)	Maternal Weight GD18^b^ (g)	No. of uterine implants	Total no. of Pups per litter^c^ (live + dead)	No. of live pups per litter^c^	% Litter loss^d^	Pregnancy rate^e^ (%)
WT	0	14	10.8 ± 0.98	34.4 ± 0.8	8.5 ± 0.6	7.1 ± 0.56	6.8 ± 0.70	14.3	53.8
	0.83	11	12.8 ± 0.81	35.0 ± 1.0	8.5 ± 0.6	6.8 ± 0.70	6.1 ± 0.82	9.1	47.8
	1.1	12	10.8 ± 0.57	33.6 ± 0.4	7.3 ± 0.5	5.6 ± 0.50	3.7 ± 0.37*	16.7	35.3
	1.5	14	12.6 ± 0.50	36.0 ± 0.5	8.4 ± 0.6	6.2 ± 0.54	4.7 ± 0.67	7.1	38.9
	2.0	17	13.2 ± 0.74	35.9 ± 0.9	7.8 ± 0.6	5.2 ± 0.54	3.1 ± 0.73**	35.3	47.2

KO	0	18	12.0 ± 0.49	35.9 ± 0.6	8.9 ± 0.4	7.8 ± 0.36	7.0 ± 0.41	11.1	75.0
	0.83	13	11.4 ± 0.73	34.6 ± 0.8	8.4 ± 0.6	7.2 ± 0.58	7.0 ± 0.54	7.7	65.0^†^
	1.1	14	11.5 ± 0.56	35.6 ± 0.6	9.2 ± 0.4	8.3 ± 0.49	7.8 ± 0.43	7.1	58.3^†^
	1.5	9	12.1 ± 0.87	35.5 ± 1.3	9.8 ± 0.6	8.5 ± 0.80	8.4 ± 0.84	11.1	20.9^†^
	2.0	16	11.0 ± 0.80	33.4 ± 0.9	8.1 ± 0.7	6.6 ± 0.62	6.4 ± 0.66	12.5	43.2^†^

Values are means ± SEM.

KO: PPAR*α* knockout; WT: wild type; GD: gestational day.

^a^Pregnancy verified by presence of uterine implantation sites.

^b^Excludes adult females not pregnant or with full litter resorption.

^c^Number of pups on day of birth at first observation.

^d^Litter loss: uterine implants present but no pups (full litter resorption) or only dead pups (whole litter loss) at birth.

^e^Pregnancy rate: (# pregnant / # plug +)∗100.

*P<.05, **P<  .001 by Bonferroni's test. ^†^ *P* < .001 by chi-square test for trend.

**Table 2:** Birth weights of wild type and PPAR*α* KO mouse pups after in utero exposure to PFNA on GD 1–18.

	Dose (mg/kg/day)	*n*	Male weight (g)	*n*	Female weight (g)
WT	0	11	1.28 ± 0.03	12	1.26 ± 0.03
	0.83	8	1.28 ± 0.03	10	1.30 ± 0.05
	1.1	10	1.29 ± 0.06	10	1.34 ± 0.06
	1.5	11	1.33 ± 0.03	12	1.32 ± 0.03
	2.0	9	1.41 ± 0.06	8	1.30 ± 0.05

KO	0	16	1.24 ± 0.03	16	1.19 ± 0.02
	0.83	12	1.28 ± 0.03	12	1.25 ± 0.03
	1.1	12	1.25 ± 0.04	13	1.20 ± 0.03
	1.5	8	1.20 ± 0.04	8	1.15 ± 0.04
	2.0	10	1.26 ± 0.03	14	1.29 ± 0.05

Values are litter means ± SEM. *n*: no. of litters. WT: wild type; KO: PPAR*α* knockout; *n*: number of litters.

**Table 3:** Liver and body weights (grams) of wild type and PPAR*α*-KO adult females and pups at necropsy (PND 21) after exposure to PFNA on GD 1–18.

Strain	Dose (mg/kg/ day)	Adult females (NP)		Adult females (P)		Pups (sexes combined)	
		Liver Weight	Body Weight	Liver Weight	Body Weight^a^	Liver Weight	Body Weight
	0	0.86 ± 0.03	22.7 ± 0.42	1.52 ± 0.87	27.3 ± 0.58	0.381 ± 0.02	9.62 ± 0.36
	0.83	1.49 ± 0.04**	22.9 ± 0.48	1.65 ± 0.07	26.4 ± 0.56	0.551 ± 0.03**	9.79 ± 0.30
WT	1.1	1.80 ± 0.04**	24.0 ± 0.43	1.91 ± 0.10*	26.2 ± 0.72	0.649 ± 0.05**	10.34 ± 0.61
	1.5	1.86 ± 0.05**	23.0 ± 0.56	2.24 ± 0.05**	28.2 ± 0.66	0.608 ± 0.02**	9.47 ± 0.23
	2.0	2.18 ± 0.04**	24.2 ± 0.36	2.51 ± 0.07**	27.5 ± 0.70	0.518 ± 0.01**	7.56 ± 0.42**
	0	0.99 ± 0.04	24.8 ± 0.74	1.88 ± 0.06	29.1 ± 0.32	0.417 ± 0.01	9.35 ± 0.19
	0.83	1.20 ± 0.04	24.7 ± 1.00	1.77 ± 0.06	28.3 ± 0.38	0.421 ± 0.01	9.16 ± 0.28

KO	1.1	1.17 ± 0.07	23.2 ± 0.54	2.02 ± 0.05	29.6 ± 0.40	0.429 ± 0.02	9.18 ± 0.26
	1.5	1.45 ± 0.04**	23.8 ± 0.41	1.74 ± 0.16	26.2 ± 1.32*	0.422 ± 0.02	8.51 ± 0.34
	2.0	1.53 ± 0.04**	23.4 ± 0.28	1.96 ± 0.10	28.0 ± 0.77	0.489 ± 0.03	8.98 ± 0.40

Values are means ± SEM.

Pup weights were on 2 pups per litter. NP: not pregnant; P: pregnant; WT: wild type; KO: PPAR*α* knockout; Wt: weight.

NP includes those with full litter resorption and no weight gain; P includes those who gave birth whether pups were live or dead.

^a^ *P* < .05 compared to NP females body weight. *P < .05, **P < .001 compared to controls within column and strain. See text for other comparisons.

**Table 4:** Serum PFNA concentrations at weaning in PPAR*α*-KO and WT Adult female mice and offspring exposed to PFNA on GD 1–18.

Strain	Dose (mg/kg/day)	Adult females with no live pups		Adult females with live pups		Pups (sexes combined)	
		*n*	PFNA (*μ*g/ml)	*n*	PFNA (*μ*g/ml)	*n* litters	PFNA (*μ*g/ml)
	0	14	0.067 ± 0.005	12	0.022 ± 0.004	9	0.033 ± 0.008
	0.83	13	28.5 ± 1.22^a^	10	8.91 ± 1.51^a^	8	9.60 ± 9.37^a^
WT	1.1	26	39.7 ± 1.26^a^	10	23.2 ± 2.57^a^	5	15.7 ± 1.42^a^
	1.5	23	48.4 ± 1.54^a^	13	21.0 ± 3.01^a^	10	17.5 ± 1.15^a^
	2.0	26	64.0 ± 2.46^a^	11	35.3 ± 3.90^a^	7	25.3 ± 2.70^a^

	0	9	0.048 ± 0.008	16	0.016 ± 0.001	16	0.068 ± 0.027
	0.83	8	38.4 ± 2.34^a^	11	2.76 ± 0.172^a^	12	15.2 ± 1.01^a^
KO	1.1	11	53.9 ± 2.51^a^	13	4.17 ± 0.310^a^	12	19.4 ± 0.69^a^
	1.5	37	72.1 ± 2.91^a^	8	11.8 ± 5.71^a^	7	26.4 ± 1.39^a^
	2.0	23	83.4 ± 2.93^a^	15	22.6 ± 5.69^a^	12	38.4 ± 1.80^a^

Values are means ± SEM or litter means ± SEM. Serum was collected from all adult females and from 2 pups per litter at 23 days post dose.

WT: wild type; KO: PPAR*α* knockout.

^a^ Significantly different from control values by *P* < .0001. See text for more statistical comparisons.
